# Minimum habitat thresholds required for conserving mountain lion genetic diversity

**DOI:** 10.1002/ece3.6723

**Published:** 2020-09-01

**Authors:** Justin A. Dellinger, Kyle D. Gustafson, Daniel J. Gammons, Holly B. Ernest, Steven G. Torres

**Affiliations:** ^1^ California Department of Fish and Wildlife Sacramento California USA; ^2^ Department of Biological Sciences Arkansas State University Jonesboro Arkansas USA; ^3^ Wildlife Genomics and Disease Ecology Laboratory University of Wyoming Laramie Wyoming USA

**Keywords:** allelic richness, effective population size, habitat use, heterozygosity, *Puma concolor*

## Abstract

Jointly considering the ecology (e.g., habitat use) and genetics (e.g., population genetic structure and diversity) of a species can increase understanding of current conservation status and inform future management practices. Previous analyses indicate that mountain lion (*Puma concolor*) populations in California are genetically structured and exhibit extreme variation in population genetic diversity. Although human development may have fragmented gene flow, we hypothesized the quantity and quality of remaining habitat available would affect the genetic viability of each population. Our results indicate that area of suitable habitat, determined via a resource selection function derived using 843,500 location fixes from 263 radio‐collared mountain lions, is strongly and positively associated with population genetic diversity and viability metrics, particularly with effective population size. Our results suggested that contiguous habitat of ≥10,000 km^2^ may be sufficient to alleviate the negative effects of genetic drift and inbreeding, allowing mountain lion populations to maintain suitable effective population sizes. Areas occupied by five of the nine geographic–genetic mountain lion populations in California fell below this habitat threshold, and two (Santa Monica Area and Santa Ana) of those five populations lack connectivity to nearby populations. Enhancing ecological conditions by protection of greater areas of suitable habitat and facilitating positive evolutionary processes by increasing connectivity (e.g., road‐crossing structures) might promote persistence of small or isolated populations. The conservation status of suitable habitat also appeared to influence genetic diversity of populations. Thus, our results demonstrate that both the area and status (i.e., protected or unprotected) of suitable habitat influence the genetic viability of mountain lion populations.

## Graphical abstract

We report a strong relationship between amount of habitat and genetic diversity in a charismatic umbrella species. We also demonstrate habitat thresholds needed to maintain genetic integrity of the species. Our work defines current conservation needs and adds to general ecological theory concerning genetic diversity and amount of available habitat.

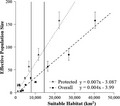

## 1 INTRODUCTION

Wildlife ecologists seek to understand wildlife habitat use patterns to preserve suitable habitat, maintain connectivity between patches of suitable habitat, and develop protocols for estimating population sizes (Proffitt et al., 2015; Smith, Duaneb, & Wilmersb, 2019; Torres, Mansfield, Foley, Lupo, & Brinkhaus, 1996; Wilmers et al., 2013; Zeller, Vickers, Ernest, & Boyce, 2017). The availability and distribution of habitat can have lasting evolutionary consequences via influences on genetic diversity (McRae, Beier, Dewald, Huynh, & Keim, 2005; Wang, Yang, Bridgman, & Lin, 2008). For example, habitat loss, fragmentation, and decreased connectivity can reduce gene flow, and promote genetic drift, genetic differentiation, and inbreeding (Delaney, Riley, & Fisher, 2010; Dixon et al., 2007; Epps et al., 2005). Thus, management planning may benefit from examining population genetics and habitat use patterns in tandem. Such an approach may support determination of minimum thresholds of habitat area that are necessary for maintaining the effective population size and thereby the evolutionary potential of wildlife populations (Caballero, Rodríguez‐Ramilo, Ávila, & Fernández, 2010; Roffler et al., 2016; Shafer et al., 2012).

Large carnivore species, which typically exist at low densities and occupy large home ranges, are some of the more challenging species to conserve, in part because of their requirements for large areas of habitat (Ray, Redford, Robert, & Berger, 2005). Such species can be used as “umbrella species” for biodiversity conservation efforts, because retention of tracts of suitable habitat large enough to support large carnivore populations benefits other species with overlapping requirements (Linnell, Swenson, & Andersen, 2000). Mountain lions (*Puma concolor*), for example, are regularly used as an umbrella species for conservation efforts across the western United States (Carroll, Noss, & Paquet, 2001; Thorne, Cameron, & Quinn, 2006). California contains large areas of suitable mountain lion habitat, totaling 165,350–170,085 km^2^ (Dellinger, Darby, & Torres, 2019), with mountain lions present in all major ecoregions (Dellinger et al., 2018, 2019; McClanahan, Duplisea, Dellinger, & Kenyon, 2017; Vickers et al., 2015; Wilmers et al., 2013). However, increasing human population size and development, particularly major roadways, has caused suitable habitat to become fragmented in multiple areas, particularly in and adjacent to the Los Angeles basin, where mountain lion populations inhabiting geographically close mountain ranges (~100 km apart) (a) are genetically distinct from each other (Gustafson et al., 2019; Morrison & Boyce, 2009; Riley et al., 2014); and (b) have low genetic diversity and effective population sizes, which could influence population viability (Benson et al., 2016, 2019; Gustafson, Vickers, Boyce, & Ernest, 2017). In contrast, other mountain lion populations in California, such as those inhabiting the Sierra Nevada Mountains and the Modoc Plateau in northeast California, are effectively large, genetically diverse, and well connected to each other with dispersal and genetic structuring influenced more by natural landscape features (e.g., the crest of the Sierra Nevada Mountains and transitions from one ecoregion to another) than by anthropogenic influences. Gustafson et al. (2019) suggested that variation in the amount of suitable habitat between populations in part explained these divergent circumstances.

To test this and related hypotheses, we examined the relationships of population genetic metrics (i.e., expected heterozygosity, allelic richness, and effective population size) for 9 genetically distinct mountain lion populations in California with: (a) the area of overall suitable habitat, estimated from a resource selection function (RSF) model that was developed using location data from GPS‐collared mountain lions, and (b) a subset of overall suitable habitat comprised of public lands and private lands with conservation easements, hereafter referred to as protected suitable habitat, where each population occurs. We hypothesized that increasing areas of suitable mountain lion habitat would be correlated with higher levels of genetic diversity (Gustafson et al., 2019; Templeton, Shaw, Routman, & Davis, 1990; Weckworth et al., 2013). We hypothesized a similar relationship between areas of protected suitable habitat and genetic diversity, but expected a given area of protected suitable habitat to be correlated with greater genetic diversity than an equally sized area of overall suitable habitat, because protected lands often have less human presence and less human‐caused disturbance than overall suitable habitat and may be perceived by mountain lions to be of higher quality (Watson, Dudley, Segan, & Hockings, 2014). In addition, based on our habitat–genetic metrics models, we identified the minimum habitat area (for both overall suitable habitat and protected suitable habitat) expected for mountain lion populations to maintain an effective population size (*N_e_*) > 50, a size where the probability of inbreeding depression is expected to be low (Franklin, 1980).

## 2 MATERIALS AND METHODS

### 2.1 Genetic analyses

**Figure 1:**
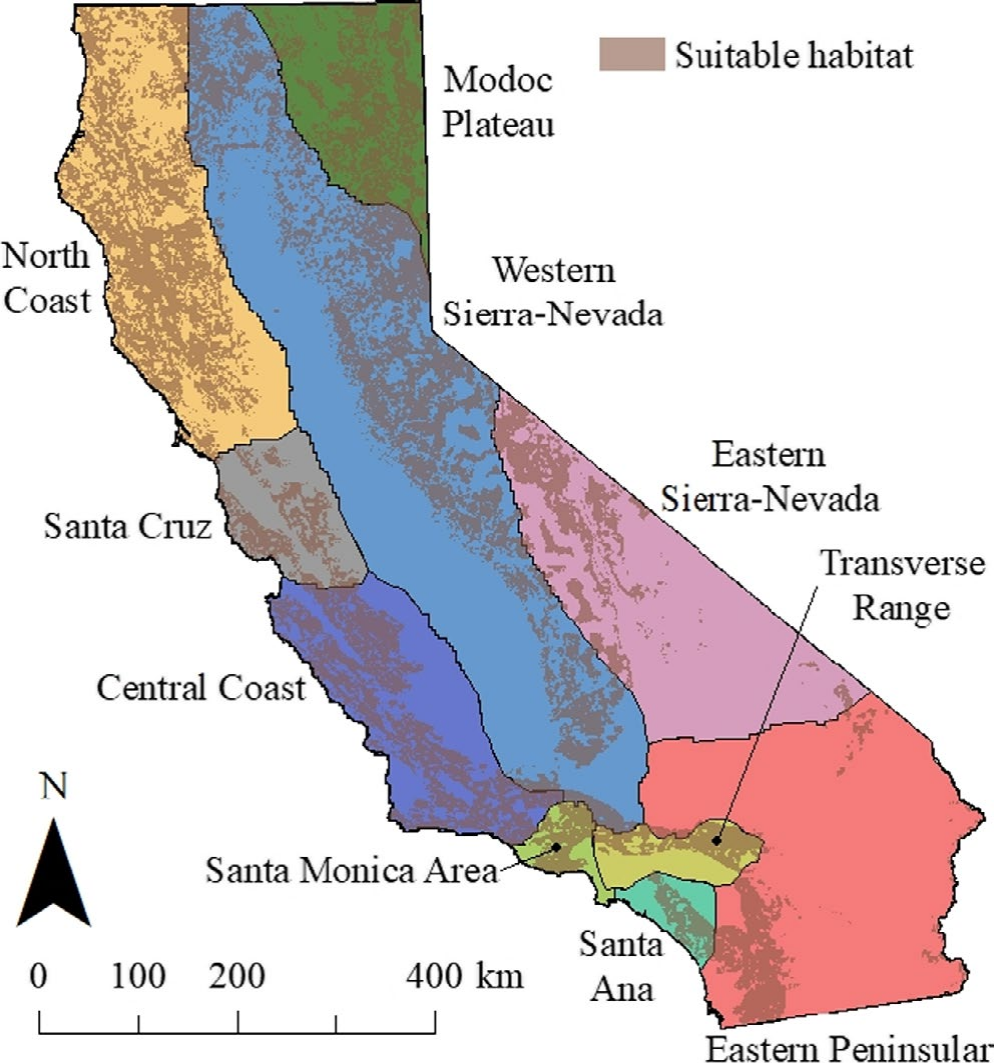
Geographic distributions of genetically distinct mountain lion populations in California (Gustafson et al., 2019) and distribution of suitable mountain lion habitat (Dellinger et al., 2020). Geographic extent of genetically distinct mountain lion populations was derived from Gustafson et al. (2019) wherein their 0–1 probability of population assignment tests were adjusted such that an area with a population assignment value ≥ 0.5 was assigned to the given genetically defined population and values < 0.5 were not. Note that there were no areas where multiple populations had population assignment values ≥ 0.5 and that population assignment values ≥ 0.5 for a given area does not inherently mean that area is suitable mountain lion habitat. Given that mountain lions in the Modoc Plateau were shown to be a genetic extension of mountain lions in Nevada, and we were unable to calculate area of suitable mountain lion habitat in Nevada, we excluded the Modoc Plateau from our analyses

We used data from two previous publications: Gustafson et al. (2019) and Dellinger et al. (2020). Gustafson et al. (2019) examined mountain lion population genetic structure using 42 microsatellite loci among 992 samples from individuals distributed throughout California and Nevada, USA. Their results demonstrated the presence of 10 geographically distinct, genetically structured mountain lion populations within California (Figure 1). We created a Geographic Information System (GIS) shapefile representing the extent and distribution of 9 of these genetically structured populations. The Modoc Plateau population was excluded because it primarily occurred in Nevada and we were unable to calculate the area of suitable mountain lion habitat outside of California (see below for habitat area calculation methods).

The spatial distribution of the 9 genetically distinct populations was determined by taking all the genetic population assignment results from the 992 samples analyzed by Gustafson et al. (2019) and performing a kriging procedure in a GIS environment. The 9 resulting probability raster layers (with values ranging from 0 to 1), one for each genetically distinct population, represented the percent likelihood that a given area belonged to each of the genetically defined mountain lion populations. We then reclassified each probability surface wherein areas with values ≥0.5 were assigned to the given genetically defined population and each area with a value <0.5 was not. We used the estimates of heterozygosity, allelic richness, and *N_e_* for each of the genetically structured populations identified by Gustafson et al. (2019; Table 1). Locus‐specific information and analyses are detailed in Gustafson et al. (2019). Briefly, specific tests for violations indicated there was no evidence for null alleles, allelic dropout, scoring errors, deviations from HWE, or linkage among loci. Data for monomorphic loci within each population were removed prior to analyses. Effective population size was estimated using the linkage disequilibrium method in NeEstimator 2.01 (Do et al., 2014). Expected heterozygosity was calculated using GenAlEx 6.502 (Peakall & Smouse, 2006, 2012), and sample‐size corrected allelic richness was calculated using FSTAT 2.9.3.2 (Goudet, 1995).

**Table 1:** Overall genetic and habitat values by area in California. Genetic values for each area are derived from Gustafson et al. (2019), while habitat‐related values for each area are derived from Dellinger et al. (2020). Overall habitat was defined as all suitable mountain lion habitat in a given area regardless of protection status. Protected habitat was defined as suitable habitat in a given area not likely to be developed in the near future (i.e., public lands or private lands with conservation easements)

Population	Expected heterozygosity	Allelic richness	Effective population size	Overall habitat (km^2^)	Protected habitat (km^2^)
Western Sierra Nevada	0.52	3.63	158 (141–177)^a^	40,397	22,183 (0.55)^b^
Eastern Sierra Nevada	0.53	3.46	23 (21–25)	10,241	9,889 (0.97)
North Coast	0.41	3.06	83 (71–97)	27,091	11,624 (0.43)
Santa Cruz	0.42	2.62	17 (15–18)	5,042	1,818 (0.36)
Central Coast	0.46	3.00	57 (47–69)	16,355	6,780 (0.41)
Santa Monica Area	0.41	2.63	3 (2–4)	2,688	1,129 (0.42)
Santa Ana	0.33	2.27	16 (13–19)	2,054	1,081 (0.53)
Eastern Peninsular	0.44	3.07	32 (29–34)	7,683	4,777 (0.62)
Transverse Range	0.42	2.75	5 (3–6)	3,759	2,976 (0.79)

^a^ Parametric 95% confidence intervals given in parentheses.

^b^ Percent of overall habitat in a given area that is protected.

### 2.2 Habitat analyses

**Figure 2:**
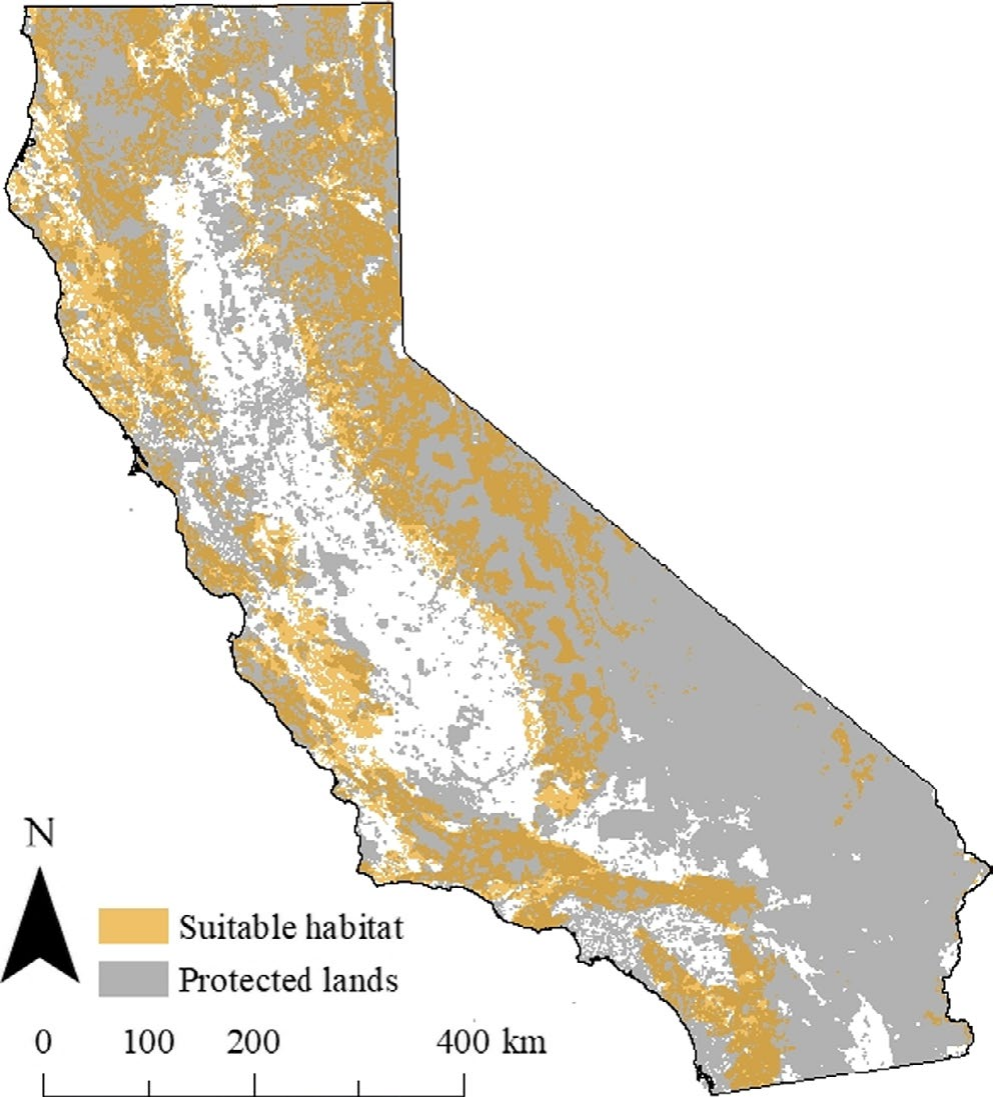
Geographic distribution of suitable mountain lion habitat (Dellinger et al., 2020) and protected lands in California. Suitable mountain lion habitat was transformed from a probability surface with values ranging from 0 to 1 to a surface with values 0 (unsuitable) or 1 (suitable) using the cut‐point probability that captured 90% of the observed mountain lion GPS data as described in Dellinger et al. (2020). Protected lands were those not likely to be developed in the near future (e.g., public lands or private lands with conservation easements)

Dellinger et al. (2020) examined mountain lion habitat use using location data from 263 GPS‐collared individuals from all major ecoregions (*n* = 8) in California (Level III Ecoregions; Griffith et al., 2016) and estimated the area of suitable habitat across the state at multiple spatial scales. We intersected a raster layer representing their resource selection function (RSF) of second‐order or home‐range level habitat selection (1 km^2^ resolution; Johnson, 1980) with the shapefile derived from Gustafson et al. (2019; Figure 1). The RSF raster layer was transformed from a probability surface with values ranging from 0 to 1 to a surface with values 0 (unsuitable) or 1 (suitable) using the cut‐point probability that captured 90% of the observed mountain lion GPS data as described in Dellinger et al. (2020). This procedure resulted in 9 individual raster layers representing area of overall suitable habitat available to each of the genetically defined mountain lion populations. We then intersected each of the 9 resulting raster layers above with a shapefile depicting protected areas (California Conservation Easement Database, 2018; California Protected Areas Database, 2018) across California, resulting in 9 additional raster layers representing the area of protected habitat available to each of the genetically defined mountain lion populations (Figure 2; Table 2).

**Table 2:** Relationships between overall (i.e., including unprotected/mixed protection status) and protected (i.e., public lands and private lands with conservation easements) suitable mountain lion habitat and each of three genetic measures: (1) expected heterozygosity, (2) allelic richness, and (3) effective population size. Logarithmic regression models were used for expected heterozygosity and allelic richness and linear regression models for effective population size. The minimum thresholds for overall and protected mountain lion habitat were derived using linear equations wherein overall and protected habitat were independent variables and effective population size was the dependent variable. We substituted *N_e_* = 50 for *x* in the linear equations

	Expected heterozygosity		Allelic richness		Effective population size	
	Overall	Protected	Overall	Protected	Overall	Protected
*R* ^2^ value	0.45	0.60	0.71	0.87	0.97	0.88
*p* value	.048	.015	.004	<.001	<.001	<.001
Minimum Threshold (km^2^)					14,591	7,923

After calculating the area of total suitable habitat and protected habitat available to each of the 9 mountain lion populations, we used logarithmic regression to evaluate the relationships between both total suitable habitat area and protected habitat area and the genetic diversity metrics (i.e., heterozygosity and allelic richness), and used linear regression to evaluate relationships between habitat areas and *N_e_* (Table 1). We used logarithmic regression for heterozygosity and allelic richness due to the differences in order of magnitude between the habitat and genetic estimates being used in the analyses. We used likelihood‐ratio tests to compare each set of models to determine whether the area of protected habitat, or overall suitable habitat was significantly better than the other in accounting for the variability in each of the three genetic metrics being examined. Lastly, using a minimum *N_e_* threshold of 50 with the linear regression equations, we derived minimum habitat thresholds predicted to be necessary for ensuring genetic population viability for each genetically defined mountain lion population (Frankham, 1995; Mace et al., 2008).

We used Program R version 3.4.3 (R Core Team, 2017) for all statistical analyses and ArcView GIS version 10.3.1 (ESRI, Redlands, California) for spatial analyses. In all analyses, we considered *p* ≤ .05 to be statistically significant.

## 3 RESULTS

The area of overall suitable habitat occupied by each of the 9 geographically and genetically distinct mountain lion populations in California varied from 2,054 km^2^ for the Santa Ana population to 40,397 km^2^ for the Western Sierra Nevada population. The proportion of suitable habitat that was protected varied from 0.36 for the Santa Cruz population to 0.97 for the Eastern Sierra Nevada population (Table 1).

**Figure 3:**
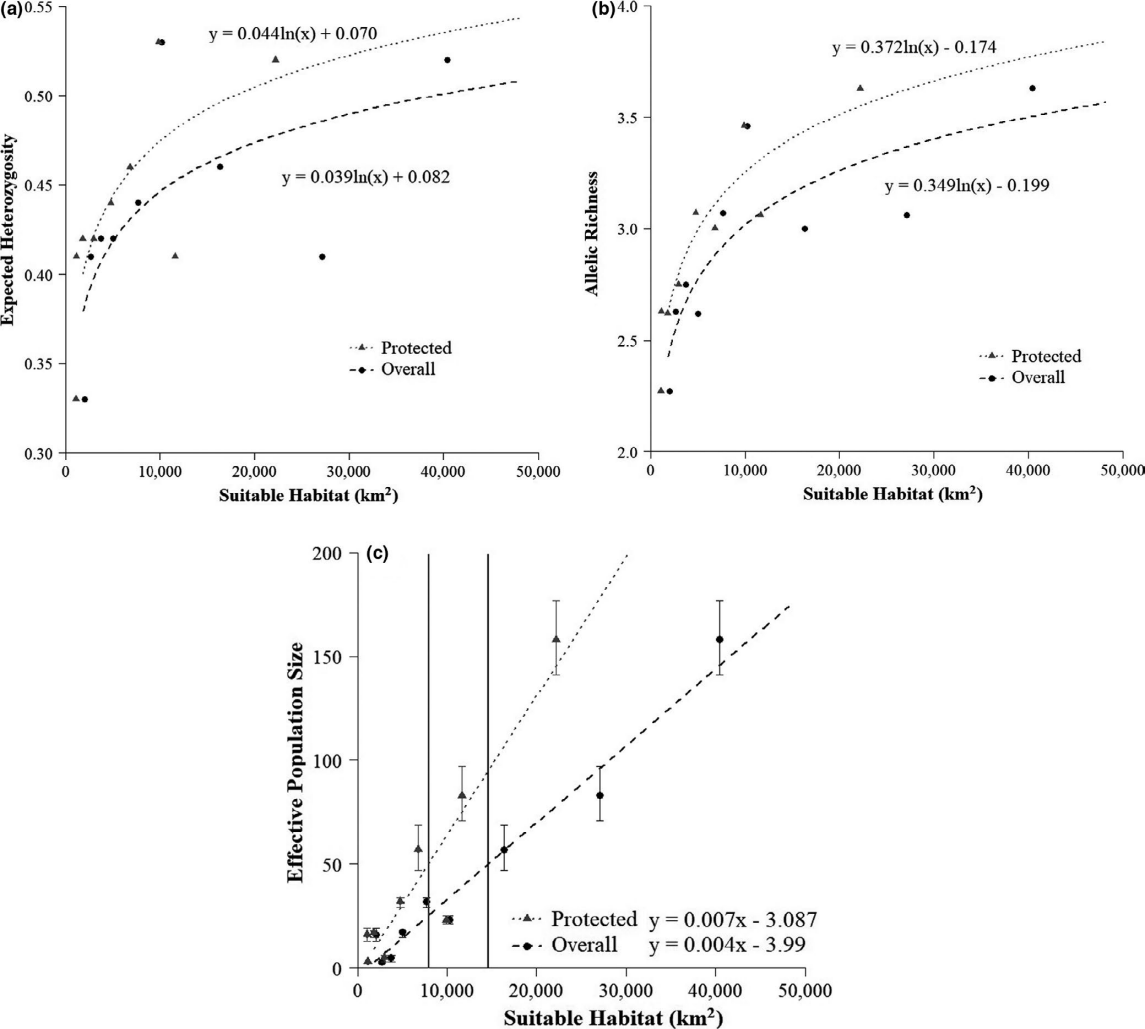
Expected heterozygosity (a), allelic richness (b), and effective population size with associated parametric 95% confidence intervals (c) of mountain lions as functions of the amount of suitable habitat (km^2^) in California. Regression lines demonstrate the relationship between overall (dashed line) or protected (dotted) suitable habitat, and a given genetic metric. Equations for each line are presented. Minimum protected (gray) and overall (black) suitable habitat thresholds needed to maintain *N_e_* ≥ 50 are displayed (c)

We observed a consistent pattern indicating that larger areas of overall suitable habitat or of protected suitable habitat occupied by the mountain lion populations were highly correlated with greater values of mountain lion conservation genetic metrics (Figure 3a‐c). For both expected heterozygosity and allelic richness, the pattern was asymptotic—the rate of increase in genetic diversity in concert with habitat variables was initially rapid but declined with additional habitat beyond ~20,000 km^2^ (Figure 3a, b). The relationship between *N_e_* and the habitat variables was linear and did not indicate the occurrence of diminishing returns with additional habitat.

We observed statistically significant relationships between all of the population genetic metrics (i.e., expected heterozygosity, allelic richness, and *N_e_*) and areas of overall and protected habitat (Table 2). The area of protected suitable habitat was a good to excellent predictor of expected heterozygosity (*r* ^2^ = 0.60) and allelic richness (*r* ^2^ = 0.87), and in both cases explained more variation that did the area of overall suitable habitat (Table 2). In contrast, while the area of protected suitable habitat was an excellent predictor of *N_e_* (*r* ^2^ = 0.88), the area of overall suitable habitat had even more predictive power (*r* ^2^ = 0.97). Likelihood‐ratio tests demonstrated that the logarithmic regression model using area of protected habitat had a significantly better fit (i.e., higher likelihood) for the given data than the same model using area of overall habitat for allelic richness (*p* = .029; $x_{2}^{2}$ = 7.042). Likelihood‐ratio tests demonstrated there was no significant difference (*p* = .245; $x_{2}^{2}$ = 2.809) between the two logarithmic regression models for expected heterozygosity, although the model using area of protected habitat had higher likelihood (17.1) than the model using area of overall habitat (15.7). Further, a likelihood‐ratio test demonstrated that the linear regression model using area of overall habitat had significantly better fit for the given data than the same model using area of protected habitat for *N_e_* (*p* = .004; $x_{2}^{2}$ = 11.138). Lastly, the minimum area of suitable habitat estimated to maintain *N_e_* ≥ 50 was 14,591 km^2^ for overall suitable habitat and 7,923 km^2^ for protected suitable habitat.

Five populations were shown to occupy areas with less than the overall and protected suitable habitat thresholds stated above. These populations were Santa Cruz, Santa Monica Area, Santa Ana, Transverse Range, and Eastern Peninsular. The Central Coast population was shown to occupy an area above the overall suitable habitat threshold but below the protected suitable habitat threshold. The Eastern Sierra Nevada population was shown to occupy an area below the overall suitable habitat threshold but above the protected suitable habitat threshold. Finally, the North Coast and Western Sierra Nevada populations were shown to occupy areas above both thresholds (Tables 1 and 2).

## 4 DISCUSSION

We found that a substantial amount of the variation in heterozygosity, allelic richness, and *N_e_* between California mountain lion populations could be explained by both the area of overall suitable habitat and the area of protected suitable habitat available. In particular, estimated *N_e_* could be predicted with knowledge of the area of overall suitable habitat. Because the area of overall suitable habitat explained 97% of the variation in *N_e_* among California mountain lion populations, it is likely in general that maintenance or restoration of any suitable habitat, protected or unprotected, would have substantial value in conserving this potential umbrella species, as well as other species with overlapping habitat requirements. For example, old‐growth redwoods (*Sequoia sempervirens*), steelhead salmon (*Oncorhynchus mykiss*), and California condors (*Gymnogyps californianus*) may fall under the umbrella and benefit from conservation of mountain lion habitat (Perrig, Donadio, Middleton, & Pauli, 2017; Thorne et al., 2006). While suitable mountain lion habitat that is protected appears to be of somewhat greater value, depending on the metric, for the genetic health of mountain lions (see below), unprotected but otherwise suitable habitat (e.g., working ranchlands, forests managed for timber production, etc.) is clearly of importance (Gray & Teels, 2006; Macon, 2020).

We also found that a given area of protected suitable habitat was correlated with higher expected heterozygosity and allelic richness than an equally sized area of overall suitable habitat. However, expected heterozygosity for a given area of protected suitable habitat was not significantly higher than an equally sized area of overall suitable habitat. It appears that if suitable habitat is protected, only about half (53%) as much is required to maintain a mountain lion population with allelic richness ≥ 3.00 than if suitable, mixed‐status (i.e., protected and unprotected) habitat is maintained. High allelic richness in protected habitat compared to unprotected/mixed‐status habitat is likely a result of less habitat fragmentation and higher landscape permeability, which can increase facilitate gene flow (Gustafson et al., 2019). Greater gene flow, combined with increased mountain lion densities, demonstrates the potential for protected habitat to serve as sources for adjacent unprotected/mixed‐status habitat and allowing for viable metapopulations of mountain lions (Mills, 2013; Zanon‐Martinez et al., 2016).

Estimates of suitable habitat available to mountain lions in California are between 165,350 km^2^ in winter and 170,085 km^2^ in summer (Dellinger et al., 2020). Although California likely contains more suitable mountain lion habitat than other states with mountain lion populations (Dickson, Roemer, McRae, & Rundall, 2013; Oregon Department of Fish & Wildlife, 2017; Robinson et al., 2015; Utah Division of Wildlife Resources, 2015), we found that 5 of the 9 genetically defined mountain lion populations in California (Eastern Peninsular, Santa Ana, Santa Cruz, Santa Monica Area, and Transverse Range) occupy areas with less overall and protected suitable habitat than our models indicate is necessary to maintain genetic diversity and adaptive potential over the long‐term. The other 3 populations (Western Sierra Nevada, Eastern Sierra Nevada, and North Coast) and likely the Modoc Plateau population, which was excluded from this analysis but is effectively large (*N_e_* = 166) and genetically diverse (Gustafson et al., 2019), contain abundant overall and protected suitable habitat.

For the 5 populations that we identified as occupying areas that were below the minimum habitat thresholds, even if all the suitable habitat within these areas was protected, our results suggest that there is insufficient habitat available to maintain long‐term genetic diversity and adaptive potential. This finding suggests that efforts to increase connectivity between these populations will likely provide more conservation benefit than those that focus on minimizing individual mortalities, particularly in light of the high rates of intraspecific strife documented in some of these populations (Benson, Sikich, & Riley, 2020; Riley et al., 2014) which indicate that remaining suitable habitat is likely saturated, providing no option for increasing abundance within each population.

Populations with below‐threshold habitat areas and low *N_e_*, however, might still be able to persist and maintain viable levels of genetic diversity if they experience gene flow with adjacent effectively large and genetically diverse populations. Greater landscape permeability would promote gene flow among distinct populations, potentially allowing for viable metapopulations of mountain lions (Mills, 2013; Olivieri, Michalakis, & Gouyon, 1995; Warren, Wallin, Beausoleil, & Warheit, 2016). Thus, single small areas of habitat with high connectivity to larger areas of habitat, as well as multiple small areas of habitat all highly connected to one another, might collectively contain enough suitable habitat to maintain a viable metapopulation (Mills, 2013; Primack, 2004). If connectivity between the Central Coast population and either the Santa Monica Area or the Santa Cruz population were restored, then the two smaller populations would then be above the habitat thresholds we have identified (Tables 1 and 2). However, if connectivity among the Transverse Range, Eastern Peninsular, and Santa Ana populations were restored, these areas combined would still fall below the habitat thresholds (Tables 1 and 2). This suggests that for mountain lions to persist in these areas, the current populations must be connected to larger source populations in the western Sierra Nevada, or maybe Mexico, and that multiple connectivity measures are required for the Santa Ana population to persist. While it is possible that mountain lions within the Eastern Peninsular population are in fact connected with lions in Arizona and/or Mexico, this region contains vast stretches of low‐quality Mojave Desert habitat in the east near toward Arizona with low mountain lion occupancy (Dellinger et al., 2019). Also, there has been little evidence of cross‐boundary connectivity from genetic or radio‐collar data from mountain lions adjacent to the Mexico border (Ernest, Vickers, Morrison, Buchalski, & Boyce, 2014). Thus, we surmised that connectivity of the Eastern Peninsular population outside of California is likely limited. The Eastern Sierra Nevada population is an example of an area with a relatively low *N_e_* and relatively small areas of suitable mountain lion habitat, yet it maintains high levels of genetic diversity, perhaps due to high levels of connectivity with adjacent large populations in Nevada and the Western Sierra Nevada (Gustafson et al., 2019), or perhaps simply legacy effects of past population genetic conditions. Our findings reinforce previous calls to focus on maintaining and improving connectivity between California mountain lion populations (Benson et al., 2016, 2019; Ernest et al., 2014; Gustafson et al., 2017, 2019; Riley et al., 2014).

Our results have particular management implications for areas such as the North Coast and Western Sierra Nevada populations that contain large areas of habitat with high genetic diversity and because of their adjacency to suitable habitat in Oregon, likely contain an unknown fraction of the true distribution, abundance, and *N_e_* of their entire respective genetically distinct populations. The Western Sierra Nevada population is an important source of genetic diversity for the rest of the state and beyond (e.g., Nevada; Gustafson et al., 2019), and it is important that this population remain connected to adjacent populations via suitable habitat. Currently, the only area connecting the Western Sierra Nevada to adjacent areas in southern and central California is the Tehachapi Mountains in Kern County. Decreased connectivity in this area likely would hasten the decline in genetic diversity of mountain lions in southern and central parts of the state. The North Coast population is essentially a peninsula connected to the Western Sierra Nevada population, and likely populations in southwest Oregon too, in the north with the southern end terminating in the North Bay area adjacent to San Francisco. Mountain lions in the southern portion of the North Coast likely can disperse only north due to large human development to the south, the Pacific Ocean to the west, and the Sacramento Valley to the east. Further, there is little protected habitat in the North Bay area (Figure 2), which could facilitate further habitat fragmentation and reduced connectivity. Thus, the southern end of the North Coast could become an eroding front for habitat and genetic variation due to potential human development of unprotected habitat and to restricted dispersal.

The Central Coast population appears to currently have adequate suitable habitat (Dellinger et al., 2020), but much of it is unprotected, making the mountain lion population along the central coast vulnerable to development and habitat loss. This area is also an important source for small and at‐risk neighboring populations (e.g., Santa Cruz and Santa Monica Area), which makes the conservation of this area essential for the viability of multiple mountain lion populations. For example, mountain lions in the Santa Cruz Mountains likely are experiencing restricted gene flow. Thus, development without consideration for conservation concerns in the Central Coast region could have major effects on connectivity and population genetics in adjacent mountain lion populations.

Across the United States, habitat loss and fragmentation are considered the most important threats to persistence of mountain lions (Cougar Management Guidelines Working Group, 2005; Hornocker & Negri, 2010) and other wildlife species (Wilcove, Rothstein, Dubow, Phillips, & Losos, 1998). These threats are in part a result of the additive effects of habitat loss and fragmentation on various aspects of wildlife ecology including, but not limited to, genetic diversity as demonstrated here with mountain lions. One way of improving the situation in various parts of California is improving habitat connectivity (e.g., wildlife road‐crossing structures) to facilitate wildlife movement and gene flow between adjacent areas (Zeller et al., 2017). One option to continue improving habitat conservation efforts is by partnering with large private landowners (e.g., ranches, timber companies) that actively work the land that they own to 1) make operations economically viable, thus keeping the land from being further degraded via increased human development; and 2) develop conservation easements to ensure that if the land is sold, it remains ecologically intact. Another option to foster habitat conservation efforts and facilitate connectivity is through interstate (e.g., Arizona, Nevada, and Oregon with respect to California) and international (e.g., Mexico with respect to California) collaborations, since wildlife populations and gene flow do not recognize political boundaries (Kark et al., 2015). Such local (i.e., private landowners) and broad (i.e., cross‐boundary) collaborations, occurring simultaneously, would help mountain lion populations in California and beyond. Both of these options likely require large collaborative efforts but working toward a common goal can dually benefit wildlife by increasing understanding of ecological processes as well as identify conservation needs. Although actions required to ensure persistence of mountain lions throughout California are challenging, protecting and improving the current area of suitable mountain lion habitat could benefit numerous other species and maintain ecosystem processes in general by ensuring the persistence of an apex predator that is integral to ecosystem function (Cougar Management Guidelines, 2005; Ripple et al., 2014; Thorne et al., 2006).

## CONFLICTS OF INTEREST

There are no conflicts of interest.

## AUTHOR CONTRIBUTION

**Justin Dellinger:** Conceptualization (lead); Data curation (equal); Formal analysis (equal); Funding acquisition (lead); Investigation (lead); Methodology (equal); Project administration (lead); Resources (lead); Software (equal); Supervision (lead); Validation (equal); Visualization (lead); Writing‐original draft (lead); Writing‐review & editing (equal). **Kyle Gustafson:** Conceptualization (equal); Data curation (equal); Formal analysis (equal); Investigation (equal); Methodology (equal); Resources (equal); Software (equal); Validation (equal); Writing‐review & editing (equal). **Daniel Gammons:** Conceptualization (supporting); Formal analysis (supporting); Investigation (supporting); Methodology (supporting); Resources (equal); Supervision (equal); Validation (equal); Writing‐review & editing (lead). **Holly Ernest:** Data curation (equal); Formal analysis (equal); Investigation (equal); Methodology (equal); Project administration (equal); Resources (equal); Supervision (equal); Validation (equal); Writing‐review & editing (equal). **Steven Torres:** Conceptualization (equal); Formal analysis (equal); Funding acquisition (lead); Investigation (equal); Project administration (equal); Resources (equal); Supervision (lead); Writing‐review & editing (lead).

## PERMITS

This study was overseen by the California Department of Fish and Wildlife (CDFW) which is the state agency in California with wildlife trustee authority. Captures led by CDFW personnel were conducted with the approval of a CDFW wildlife veterinarian and under the scope of CDFW’s animal care and use policy (CDFW Operations Manual Policy 149). Captures led by non‐CDFW personnel (e.g., universities or nongovernmental organizations) were conducted under the approval of CDFW (permit numbers SC‐011968, SC‐007303, SC‐002730, SC‐009875, and SC‐013416) or an affiliated animal care and use committee (University of California, Santa Cruz, protocol number Wilmc1101; University of California, Davis, protocol number 17233).

## Data Availability

Through agreements with nonprofit organizations, private landowners, and Native American Tribes, exact GPS coordinates of mountain lion genetic samples and radio‐collared mountain lion location data are not to be publicly shared. Spatial locations of mountain lion genetic samples are referenced to the nearest town or city, and relevant microsatellite genotypes and associated location data are available on Dryad: https://doi.org/10.5061/dryad.j76c4k4. Values of genetic metrics and suitable mountain lion habitat in each mountain lion population are available on Dryad: https://doi.org/10.5061/dryad.nvx0k6dqb. The tables within this publication contain all the numbers used for analyses herein.

## References

[ece36723-bib-0001] Benson, J. F. , Mahoney, P. J. , Sikich, J. A. , Serieys, L. E. K. , Pollinger, J. P. , Ernest, H. B. , and Riley, S. P. D. (2016). Interactions between demography, genetics, and landscape connectivity increase extinction probability for a small population of large carnivores in a major metropolitan area. Proceedings of the Royal Society B, 283, 20160957. 27581877 10.1098/rspb.2016.0957 PMC5013791

[ece36723-bib-0002] Benson, J. F. , Mahoney, P. J. , Vickers, T. W. , Sikich, J. A. , Beier, P. , Riley, S. P. D. , … Boyce, W. M. (2019). Extinction vortex dynamics of top predators isolated by urbanization. Ecological Applications, 29, e01868 10.1002/eap.1868 30892753

[ece36723-bib-0003] Benson, J. F. , Sikich, J. A. , & Riley, S. P. D. (2020). Survival and competing mortality risks of mountain lions in a major metropolitan area. Biological Conservation, 241, 108294 10.1016/j.biocon.2019.108294

[ece36723-bib-0004] Caballero, A. , Rodríguez‐Ramilo, S. T. , Ávila, V. , & Fernández, J. (2010). Management of genetic diversity of subdivided populations in conservation programmes. Conservation Genetics, 11, 409–419. 10.1007/s10592-009-0020-0

[ece36723-bib-0005] California Conservation Easement Database . (2018). www.calands.org (December 2018).

[ece36723-bib-0006] California Protected Areas Database . (2018). www.calands.org (December 2018).

[ece36723-bib-0007] Carroll, C. , Noss, R. F. , & Paquet, P. C. (2001). Carnivores as focal species for conservation planning in the Rocky Mountain region. Ecological Applications, 11, 961–980. 10.1890/1051-0761(2001)011[0961:CAFSFC]2.0.CO;2

[ece36723-bib-0008] Cougar Management Guidelines Working Group . (2005). Cougar management guidelines. Bainbridge Island, Washington: WildFutures Publishing.

[ece36723-bib-0009] Delaney, K. S. , Riley, S. P. D. , Fisher, R. N. (2010). A rapid, strong, and convergent genetic response to urban habitat fragmentation in four divergent and widespread vertebrates. PLoS One, 5, e12767. 20862274 10.1371/journal.pone.0012767 PMC2940822

[ece36723-bib-0010] Dellinger, J. A. , Cristescu, B. , Ewanyk, J. , Gammons, D. J. , Garcelon, D. , Johnston, P. (2020). Using mountain lion habitat selection in management. The Journal of Wildlife Management, 84, 359–371. 10.1002/jwmg.21798

[ece36723-bib-0011] Dellinger, J. A. , Darby, N. W. , & Torres, S. G. (2019). Factors influencing occupancy and detection rates of mountain lions in the Mojave Desert of California. The Southwestern Naturalist, 63, 248–255. 10.1894/0038-4909-63-4-248

[ece36723-bib-0012] Dellinger, J. A. , Loft, E. R. , Bertram, R. C. , Neal, D. L. , Kenyon, M. W. , Torres, S. G. (2018). Seasonal spatial ecology of mountain lions (*Puma concolor*) in the central Sierra‐Nevada Mountains. West North American Naturalist, 78, 143–156.

[ece36723-bib-0013] Dickson, B. G. , Roemer, G. W. , McRae, B. H. , & Rundall, J. M. (2013). Models of regional habitat quality and connectivity for pumas (*Puma concolor*) in the southwestern United States. PLoS One, 8, e81898 10.1371/journal.pone.0081898 24367495 PMC3867332

[ece36723-bib-0014] Dixon, J. D. , Oil, M. K. , Wooten, M. C. , Eason, T. H. , Walter McCown, J. , Cunningham, M. W. (2007). Genetic consequences of habitat fragmentation and loss: The case of the Florida black bear. Conservation Genetics, 8, 455–464.

[ece36723-bib-0015] Do, C. , Waples, R. S. , Peel, D. , Macbeth, G. M. , Tillett, B. J. , Ovenden, J. R. (2014). NeEstimator v2: Re‐Implementation of software for the estimation of contemporary effective population size (*N* *_e_*) from genetic data. Molecular Ecology Resources, 14, 209–214. 23992227 10.1111/1755-0998.12157

[ece36723-bib-0016] Epps, C. W. , Palsbøll, P. J. , Wehausen, J. D. , Roderick, G. K. , Ramey, R. R. , & McCullough, D. R. (2005). Highways block gene flow and cause a rapid decline in genetic diversity of desert bighorn sheep. Ecology Letters, 8, 1029–1038. 10.1111/j.1461-0248.2005.00804.x

[ece36723-bib-0017] Ernest, H. B. , Vickers, T. W. , Morrison, S. A. , Buchalski, M. R. , & Boyce, W. M. (2014). Fractured genetic connectivity threatens a southern California puma (*Puma concolor*) population. PLoS One, 9, e107985 10.1371/journal.pone.0107985 25295530 PMC4189954

[ece36723-bib-0018] Frankham, R. (1995). Effective population size/adult population size ratios in wildlife: A review. Genetical Research, 66, 95–107. 10.1017/S0016672300034455 18976539

[ece36723-bib-0019] Franklin, I. (1980). Evolutionary change in small populations In Soule M. E. and Wilcox B. A. (Ed.), Conservation biology: An evolutionary‐ecological perspective (pp. 135–149). Sunderland, MA: Sinauer Associates Inc.

[ece36723-bib-0020] Goudet, J. (1995). FSTAT (version 1.2): A computer program to calculate *F*‐statistics. Journal of Heredity, 86, 485–486. 10.1093/oxfordjournals.jhered.a111627

[ece36723-bib-0021] Gray, R. L. , & Teels, B. M. (2006). Wildlife and fish conservation through the Farm Bill. Wildlife Society Bulletin, 34, 906–913. 10.2193/0091-7648(2006)34[906:WAFCTT]2.0.CO;2

[ece36723-bib-0022] Griffith, G. E. , Omernik, J. M. , Smith, D. W. , Cook, T. D. , Tallyn, E. D. , Moseley, K. and Johnson, C. B. (2016). Ecoregions of California: U.S. Geological Survey Open‐File Report 2016–1021. doi: 10.3133/ofv20161021

[ece36723-bib-0023] Gustafson, K. D. , Gagne, R. B. , Vickers, T. W. , Riley, S. P. D. , Wilmers, C. C. , Bleich, V. C. … Ernest, H. B. (2019). Genetic source‐sink dynamics among naturally structured and anthropogenically fragmented puma populations. Conservation Genetics, 20, 215–227. 10.1007/s10592-018-1125-0

[ece36723-bib-0024] Gustafson, K. D. , Vickers, T. W. , Boyce, W. M. , & Ernest, H. B. (2017). A single migrant enhances the genetic diversity of an inbred puma population. Royal Society Open Science, 4, 170115 10.1098/rsos.170115 28573020 PMC5451821

[ece36723-bib-0025] Hornocker, M. , & Negri, S. (2010). Cougar: Ecology and conservation. Chicago: Univ. of Chicago Press.

[ece36723-bib-0026] Johnson, D. H. (1980). The comparison of usage and availability measurements for evaluating resource preference. Ecology, 61, 65–71. 10.2307/1937156

[ece36723-bib-0027] Kark, S. , Tulloch, A. , Gordon, A. , Mazor, T. , Bunnefeld, N. , & Levin, N. (2015). Cross‐boundary collaboration: Key to the conservation puzzle. Current Opinion in Environmental Sustainability, 12, 12–24. 10.1016/j.cosust.2014.08.005

[ece36723-bib-0028] Linnell, J. D. C. , Swenson, J. E. , Andersen, R. (2000). Conservation of biodiversity in Scandinavian boreal forests: Large carnivores as flagships, umbrellas, indicators, or keystones? Biodiversity and Conservation, 9, 857–868.

[ece36723-bib-0029] Mace, G. M. , Collar, N. J. , Gaston, K. J. , Hilton‐taylor, C. , Akçakaya, H. R. , Leader‐williams, N. , … Stuart, S. N. (2008). Quantification of extinction risk: IUCN’s system for classifying threatened species. Conservation Biology, 22, 1424–1442. 10.1111/j.1523-1739.2008.01044.x 18847444

[ece36723-bib-0030] Macon, D. (2020). Paying for presence of predators: An evolving approach to compensating ranchers. Rangelands, 42, 43–52.

[ece36723-bib-0031] McClanahan, K. A. , Duplisea, B. N. , Dellinger, J. A. , Kenyon, A. M. W. (2017). Documentation of mountain lion occurrence and reproduction in the Sacramento Valley of California. California Fish and Game, 103, 7–14.

[ece36723-bib-0032] McRae, B. H. , Beier, P. , Dewald, L. E. , Huynh, L. Y. , Keim, P. (2005). Habitat barriers limit gene flow and illuminate historical events in a wide‐ranging carnivore, the American puma. Molecular Ecology, 14, 1965–1977. 15910319 10.1111/j.1365-294x.2005.02571.x

[ece36723-bib-0033] Mills, L. S. (2013). Conservation of Wildlife Populations: Demography, Genetics, and Management, 2nd ed New York, NY: Wiley‐Blackwell Publishing.

[ece36723-bib-0034] Morrison, S. A. , & Boyce, W. M. (2009). Conserving connectivity: Some lessons from mountain lions in southern California. Conservation Biology, 23, 275–285. 10.1111/j.1523-1739.2008.01079.x 18983604

[ece36723-bib-0035] Olivieri, I. , Michalakis, Y. , & Gouyon, P.‐H. (1995). Metapopulation genetics and the evolution of dispersal. The American Naturalist, 146, 202–228. 10.1086/285795

[ece36723-bib-0036] Oregon Department of Fish and Wildlife . (2017). Oregon cougar management plan. Salem, OR: ODFW.

[ece36723-bib-0037] Peakall, R. , & Smouse, P. E. (2006). GENALEX 6: Genetic analysis in Excel. Population genetic software for teaching and research. Molecular Ecology Notes, 6, 288–295. 10.1093/bioinformatics/bts460 PMC3463245 22820204

[ece36723-bib-0038] Peakall, R. , & Smouse, P. E. (2012). GENALEX 6.5: Genetic analysis in Excel. Population genetic software for teaching and research – an update. Bioinformatics, 28, 2537–2539. 10.1093/bioinformatics/bts460 22820204 PMC3463245

[ece36723-bib-0039] Perrig, P. L. , Donadio, E. , Middleton, A. D. , & Pauli, J. N. (2017). Puma predation subsidizes an obligate scavenger in the high Andes. Journal of Applied Ecology, 54, 846–853. 10.1111/1365-2664.12802

[ece36723-bib-0040] Primack, R. B. (2004). A Primer of Conservation Biology, 3rd ed Sunderland, MA: Sinauer Associates Inc.

[ece36723-bib-0041] Proffitt, K. M. , Goldberg, J. F. , Hebblewhite, M. , Russell, R. , Jimenez, B. S. , Robinson, H. S. , … Schwartz, M. K. (2015). Integrating resource selection into spatial capture‐recapture models for large carnivores. Ecosphere, 6, 1–15. 10.1890/ES15-00001.1

[ece36723-bib-0042] R Core Team (2017). R: A language and environment for statistical computing. Vienna, Austria: R Foundation for Statistical Computing http://www.R‐project.org

[ece36723-bib-0043] Ray, J. , Redford, K. , Robert, S. , Berger, J. (2005). Large Carnivores and the Conservation of Biodiversity. Washington, D.C.: Island Press.

[ece36723-bib-0044] Riley, S. P. D. , Serieys, L. E. K. , Pollinger, J. P. , Sikich, J. A. , Dalbeck, L. , Wayne, R. K. , & Ernest, H. B. (2014). Individual behaviors dominate the dynamics of an urban mountain lion population isolated by roads. Current Biology, 24, 1989–1994. 10.1016/j.cub.2014.07.029 25131676

[ece36723-bib-0045] Ripple, W. J. , Estes, J. A. , Beschta, R. L. , Wilmers, C. C. , Ritchie, E. G. , Hebblewhite, M. , … Wirsing, A. J. (2014). Status and ecological effects of the world’s largest carnivores. Science, 343, 1241484 10.1126/science.1241484 24408439

[ece36723-bib-0046] Robinson, H. S. , Ruth, T. , Gude, J. A. , Choate, D. , DeSimone, R. , Hebblewhite, M. , … Williams, J. (2015). Linking resource selection and mortality modeling for population estimation of mountain lions in Montana. Ecological Modelling, 312, 11–25. 10.1016/j.ecolmodel.2015.05.013

[ece36723-bib-0047] Roffler, G. H. , Schwartz, M. K. , Pilgrim, K. L. , Talbot, S. L. , Sage, G. K. , Adams, L. G. , & Luikart, G. (2016). Identification of landscape features influencing gene flow: How useful are habitat selection models? Evolutionary Applications, 9, 805–817. 10.1111/eva.12389 27330556 PMC4908466

[ece36723-bib-0048] Shafer, A. B. , Northrup, J. M. , White, K. S. , Boyce, M. S. , Côté, S. D. , & Coltman, D. W. (2012). Habitat selection predicts genetic relatedness in an alpine ungulate. Ecology, 93, 1317–1329. 10.1890/11-0815.1 22834373

[ece36723-bib-0049] Smith, J. A. , Duaneb, T. P. , Wilmersb, C. C. (2019). Moving through the matrix: Promoting permeability for large carnivore in a human‐dominated landscape. Landscape and Urban Planning, 183, 50–58.

[ece36723-bib-0050] Templeton, A. R. , Shaw, K. , Routman, E. , & Davis, S. K. (1990). The genetic consequences of habitat fragmentation. Annals of the Missouri Botanical Garden, 77(1), 13–27. 10.2307/2399621

[ece36723-bib-0051] Thorne, J. H. , Cameron, D. , & Quinn, J. F. (2006). A conservation design for the central coast of California and the evaluation of mountain lion as an umbrella species. Natural Areas Journal, 26, 137–148. 10.3375/0885-8608(2006)26[137:ACDFTC]2.0.CO;2

[ece36723-bib-0052] Torres, S. G. , Mansfield, T. M. , Foley, J. E. , Lupo, T. , Brinkhaus, A. (1996). Mountain lion and human activity in California: Testing speculations. Wildlife Society Bulletin, 24, 451–460.

[ece36723-bib-0053] Utah Division of Wildlife Resources . (2015). Utah Cougar Management Plan V.3 2015–2025. Salt Lake City, UT: DWR Publication No. 15–28.

[ece36723-bib-0054] Vickers, T. W. , Sanchez, J. N. , Johnson, C. K. , Morrison, S. A. , Botta, R. , Smith, T. , … Boyce, W. M. (2015). Survival and mortality of pumas (*Puma concolor*) in a fragmented, urbanizing landscape. PLoS One, 9, e0131490. 10.1371/journal.pone.0131490 PMC4503643 26177290

[ece36723-bib-0055] Wang, Y. H. , Yang, K. C. , Bridgman, C. L. & Lin, L.‐K. (2008). Habitat suitability modelling to correlate gene flow with landscape suitability. Landscape Ecology, 23, 989–1000.

[ece36723-bib-0056] Warren, M. J. , Wallin, D. O. , Beausoleil, R. A. , & Warheit, K. I. (2016). Forest cover mediates genetic connectivity of northwestern cougars. Conservation Genetics, 17, 1011–1024. 10.1007/s10592-016-0840-7

[ece36723-bib-0057] Watson, J. E. M. , Dudley, N. , Segan, D. B. , & Hockings, M. (2014). The performance and potential of protected areas. Nature, 515, 67 10.1038/nature13947 25373676

[ece36723-bib-0058] Weckworth, B. V. , Musiani, M. , DeCesare, N. J. , McDevitt, A. D. , Hebblewhite, M. , & Mariani, S. (2013). Preferred habitat and effective population size drive landscape genetic patterns in an endangered species. Proceedings of the Royal Society B: Biological Sciences, 280, 20131756 10.1098/rspb.2013.1756 PMC3768318 24004939

[ece36723-bib-0059] Wilcove, D. S. , Rothstein, D. , Dubow, J. , Phillips, A. , & Losos, E. (1998). Quantifying threats to imperiled species in the United States. BioScience, 48, 607–615. 10.2307/1313420

[ece36723-bib-0060] Wilmers, C. C. , Wang, Y. , Nickel, B. , Houghtaling, P. , Shakeri, Y. , Allen, M. L. , … Williams, T. (2013). Scale dependent behavioral responses to human development by a large predator, the puma. PLoS One, 8, e60590 10.1371/journal.pone.0060590 23613732 PMC3629074

[ece36723-bib-0061] Zanon‐Martinez, J. I. , Kelly, M. J. , Mesa‐Cruz, J. B. , Sarasola, J. H. , DeHart, C. , & Travaini, A. (2016). Density and activity patterns of pumas in hunted and non‐hunted areas in central Argentina. Wildlife Research, 43, 449–460. 10.1071/WR16056

[ece36723-bib-0062] Zeller, K. A. , Vickers, T. W. , Ernest, H. B. , Boyce, W. M. (2017). Multi‐level, multi‐scale resource selection functions and resistance surfaces for conservation planning: Pumas as a case study. PLoS One, 12, e0179570. 28609466 10.1371/journal.pone.0179570 PMC5469479

